# Altered Cardiovascular Defense to Hypotensive Stress in the Chronically Hypoxic Fetus

**DOI:** 10.1161/HYPERTENSIONAHA.120.15384

**Published:** 2020-08-31

**Authors:** Beth J. Allison, Kirsty L. Brain, Youguo Niu, Andrew D. Kane, Emilio A. Herrera, Avnesh S. Thakor, Kimberley J. Botting, Christine M. Cross, Nozomi Itani, Caroline J. Shaw, Katie L. Skeffington, Chritian Beck, Dino A. Giussani

**Affiliations:** 1 From the Department of Physiology, Development and Neuroscience, University of Cambridge, United Kingdom (B.J.A., K.L.B., Y.N., A.D.K., E.A.H., A.S.T., K.J.B., C.M.C., N.I., C.J.S., K.L.S., C.B., D.A.G.); 2 Department of Radiology, Stanford University Medical Center, Palo Alto, CA (A.V.S.); 3 Institute of Reproductive and Developmental Biology, Imperial College, London United Kingdom (C.J.S.); 4 Cambridge Cardiovascular Strategic Research Initiative (D.A.G.); 5 Cambridge Strategic Research Initiative in Reproduction (D.A.G.).

**Keywords:** blood pressure, fetus, hypotension, pregnancy, sheep

## Abstract

The hypoxic fetus is at greater risk of cardiovascular demise during a challenge, but the reasons behind this are unknown. Clinically, progress has been hampered by the inability to study the human fetus non-invasively for long period of gestation. Using experimental animals, there has also been an inability to induce gestational hypoxia while recording fetal cardiovascular function as the hypoxic pregnancy is occurring. We use novel technology in sheep pregnancy that combines induction of controlled chronic hypoxia with simultaneous, wireless recording of blood pressure and blood flow signals from the fetus. Here, we investigated the cardiovascular defense of the hypoxic fetus to superimposed acute hypotension. Pregnant ewes carrying singleton fetuses surgically prepared with catheters and flow probes were randomly exposed to normoxia or chronic hypoxia from 121±1 days of gestation (term ≈145 days). After 10 days of exposure, fetuses were subjected to acute hypotension via fetal nitroprusside intravenous infusion. Underlying in vivo mechanisms were explored by (1) analyzing fetal cardiac and peripheral vasomotor baroreflex function; (2) measuring the fetal plasma catecholamines; and (3) establishing fetal femoral vasoconstrictor responses to the α_1_-adrenergic agonist phenylephrine. Relative to controls, chronically hypoxic fetal sheep had reversed cardiac and impaired vasomotor baroreflex function, despite similar noradrenaline and greater adrenaline increments in plasma during hypotension. Chronic hypoxia markedly diminished the fetal vasopressor responses to phenylephrine. Therefore, we show that the chronically hypoxic fetus displays markedly different cardiovascular responses to acute hypotension, providing in vivo evidence of mechanisms linking its greater susceptibility to superimposed stress.

## Abstract 2

Supplemental Digital Content is available in the text.

A reduction in fetal oxygenation or fetal hypoxia is one of the most common consequences of complicated pregnancy.^1^ Studies have long established that the developing fetus in late gestation has robust cardiovascular compensatory mechanisms to withstand an acute episode of hypoxia.^2,3^ By marked contrast, we know little about the effects of chronic hypoxia on the fetal cardiovascular system and even less during a superimposed challenge.^4–6^ Important human clinical studies have reported that the growth restricted fetus displays significant alteration in cardiovascular structure and function. However, whether these effects are mediated by chronic fetal hypoxia or other deficiencies has been difficult to isolate.^7–9^ Experimental progress in the field of hypoxic pregnancy using animal models has been hampered for several reasons. Most importantly, there has been an inability to induce hypoxia for long periods of gestation while recording cardiovascular data from the fetus. This gap of knowledge is of substantial clinical significance because the growth-restricted fetus, presumed to be chronically hypoxic, is at a 7-fold risk of acute complications later in gestation. However, the reason behind this is completely unknown.^10^

The late gestation fetus has well-described compensatory responses to alterations in blood pressure. In response to acute hypotension, cardiac and vasomotor baroreflex responses are triggered.^11,12^ Neural sympathetic outflow to arterioles increases fetal peripheral vascular resistance, and on the venous side, it promotes an increase in venous return to the fetal heart. A fall in vagal outflow and an increase in sympathetic stimulation lead to cardiac sympathetic dominant effects. This increases fetal heart rate and ventricular contractility, both of which act in concert with the vasomotor responses to restore fetal arterial blood pressure back to baseline.^11,12^ Fetal plasma catecholamines also increase in response to acute hypotension to maintain the neurally triggered cardiovascular and metabolic sympathetic responses.^11–13^

Clinical studies of heart rate variability have provided evidence to support that the growth-restricted human fetus also displays a compromised autonomic control of cardiovascular function.^14^ These studies suggest increased vulnerability to blood pressure instability, which may predispose these infants to systemic hypoperfusion, reducing oxygen and glucose delivery to the fetal brain.^8^ However, whether these effects occur because of chronic hypoxia during pregnancy, or how chronic hypoxia affects fetal cardiovascular defenses to alterations in blood pressure homeostasis and underlying mechanisms are completely unknown.

We have created isobaric hypoxic chambers able to house pregnant ewes for long periods of gestation under highly controlled chronic hypoxic conditions. We have also created a wireless data acquisition system, the CamDAS, able to record in vivo cardiovascular data from singleton fetuses being carried by free-moving pregnant ewes within the hypoxic chambers.^5,6,15^ Fetal cardiovascular data can then be transmitted by Bluetooth technology to a laptop outside the chamber (Figure S1 in the Data Supplement). In this study, we have used this system to determine in vivo the fetal cardiovascular responses to an episode of acute hypotension in the chronically hypoxic fetus. Underlying mechanisms were explored adopting a 3-pronged approach, by (1) constructing fetal in vivo cardiac and peripheral vasomotor baroreflex responses to acute hypotension; (2) measuring the in vivo fetal plasma catecholamine response to acute hypotension; and (3) establishing fetal in vivo pressor and peripheral vasopressor responses to exogenous bolus doses of the α_1_-adrenergic agonist phenylephrine. Further, to investigate the capacity of the chronically hypoxic fetus to maintain perfusion of the brain during acute hypotension, we also calculated in vivo changes in oxygen and glucose delivery in the carotid and femoral vascular beds. The study tested the hypothesis that chronic hypoxia significantly impacts on the fetal cardiovascular defense to a superimposed acute hypotensive challenge.

## Methods

### Data Availability

The data underlying this article are available in the article. Any additional data required associated with this article can be made available from the corresponding author.

### Ethical Approval

All procedures were performed at The Barcroft Centre of the University of Cambridge under the UK Animals (Scientific Procedures) Act 1986 and were approved by the Ethical Review Board of the University of Cambridge. The experimental design was conducted in accordance with the ARRIVE guidelines.

### Surgical Preparation

Animals were instrumented as previously described.^5,6,15,16^ Briefly, 12 Welsh Mountain pregnant ewes carrying singleton male fetuses were surgically instrumented at 116±1 days of gestational age (term is ca 145 days). Food but not water was withheld from the pregnant ewe for 24 hours before surgery. On the day of surgery, the ewe was transferred to a preoperative room, where the neck fleece was clipped and anesthesia was induced via injection of Alfaxan into the jugular vein (1.5–2.5 mg/kg Alfaxalone; Jurox Ltd, Worcestershire, United Kingdom). The ewe was then placed on her back and intubated (Portex cuffed endotracheal tube; Smiths Medical International, Ltd, Kent, United Kingdom). Anesthesia was maintained by spontaneous inhalation of 1.5% isoflurane in O_2_ (2 L/minute; IsoFlo; Abbott laboratories, Ltd, Berkshire, United Kingdom), and the abdomen, flanks, and medial surfaces of the hind limbs were shaved and cleaned. Antibiotics (30 mg/kg I.M. procaine benzylpenicillin; Depocillin; Intervet UK, Ltd, Milton Keynes, United Kingdom) and an analgesic agent (1.4 mg/kg S.C. carprofen; Rimadyl; Pfizer Ltd, Kent, United Kingdom) were administered.

Following transfer to the surgical theater, general anesthesia (1.5%–2.0% isoflurane in 60:40 O_2_:N_2_O) was maintained using positive pressure ventilation in a nonrebreathing circuit (Datex-Ohmeda Ltd, Hatfield, Hertfordshire, United Kingdom). The animal was covered with sterile drapes and midline abdominal, and uterine incisions were made to expose the fetal hind limbs, while minimizing loss of amniotic fluid, as described previously.^16–18^ On one side, fetal femoral arterial (i.d., 0.86 mm; o.d., 1.52 mm; Critchly Electrical Products, NSW, Australia) and venous (i.d., 0.56 mm; o.d., 0.96 mm) catheters were inserted. Another catheter was anchored onto the fetal hindlimb skin for recording of the reference amniotic pressure. A Transonic flow probe was positioned around the contra-lateral femoral artery (MC2RS-JSF-WC120-CS12-GCP, Transonic Systems Inc. Ithaca, New York). The uterine incision was closed in layers. Through a second uterotomy, the fetal head was exteriorized. The fetal carotid arteries were isolated and on one side a catheter was inserted. A second Transonic flow probe (MC2RS-JSF-WC120-CS12-GCP) was positioned around the contra-lateral carotid artery.^5,17^ The uterus was closed in layers and catheters and flow probe leads were exteriorized via keyhole incisions in the maternal flank. Catheters were exteriorized on the ewe’s right side while the flow probe leads were exteriorized through the ewe’s left flank. The maternal abdominal and skin incisions were then closed, as before.^5,6,15–19^ The maternal femoral vessels on the right leg were then isolated. A Teflon catheter (i.d. 1.0 mm, o.d. 1.6 mm, Altec, United Kingdom) was then inserted into the maternal femoral artery and placed in the descending aorta. A maternal femoral venous catheter was inserted (i.d., 0.86 mm; o.d., 1.52 mm; Critchly Electrical Products, NSW, Australia). These catheters were exteriorized through the same keyhole on the ewe’s right-side flank, and the skin incision was closed.

While under general anesthesia, the ewe was then fitted with a bespoke jacket housing the wireless Cambridge Data Acquisition System (CamDAS, Maastricht Instruments, Maastricht, The Netherlands.^5,6,15^ The catheters were connected to pressure transducers (COBE; Argon Division, Maxxim Medical, Athens, TX) within the pressure box housed in a pocket of the jacket on one side, and the flow probes leads were connected to the flow meter housed within a pocket of the jacket on the other side (Figure S1). Heart rate was triggered from the arterial blood pressure and flow pulsatile waveforms. Recordings of fetal arterial blood pressure and heart rate, amniotic pressure, as well as carotid and femoral blood flow were then continuously transmitted wirelessly via Bluetooth technology onto a laptop computer and, from this moment on, recorded continuously until post-mortem. The ewe was recovered from anesthesia and extubated when spontaneous breathing returned.^5,6,15–19^ Typically, surgery lasted just over 2 hours.

Ewes were housed in individual pens in rooms with a 12 hour:12 hour/light:dark cycle where they had free access to hay and water and were fed concentrates twice daily (100 g sheep nuts no. 6; H & C Beart Ltd, Kings Lynn, United Kingdom). Antibiotics were administered daily to the ewe (0.20–0.25 mg/kg I.M. depocillin; Mycofarm, Cambridge, United Kingdom) and the fetus intravenously and into the amniotic cavity (600 mg in 2 mL 0.9% NaCl, benzylpenicillin; Crystapen, Schering-Plough, Animal Health Division, Welwyn Garden City, United Kingdom). Generally, normal feeding patterns were restored within 24 to 48 hours after surgery. Ewes were then randomly allocated to 1 of the 2 experimental groups: normoxia (n=6) or chronic hypoxia (n=6).

### Chronic Hypoxia Protocol

Ewes allocated to chronic hypoxia were housed in 1 of 4 bespoke isobaric hypoxic chambers at The Barcroft Centre of the University of Cambridge (Telstar Ace, Dewsbury, West Yorkshire, United Kingdom).^5,6,15^ Ambient PO_2_, PCO_2_, humidity, and temperature within each chamber were monitored via sensors and values recorded continuously (Figure S1). Pregnancies assigned to the chronic hypoxia group were placed inside the chambers under normoxic conditions (11 L/second air, equating to 39.6 m^3^/hour) to acclimatize for 2 days. On the 5th postoperative day at 121±1 days of gestation, pregnancies assigned to chronic hypoxia were exposed to ca10% O_2_ by altering the incoming gas mixture to 5 L/second air: 6 L/second N_2_. Air and nitrogen provision came from a specially designed air and nitrogen generating system (Domnick Hunter Gas Generation, Gateshead, Tyne & Wear, United Kingdom^5^). Within the chambers, the induction of hypoxia was gradual, achieving 10% O_2_ over 24 hours. From this day on, chronic hypoxia was maintained at 10.0±0.05% O_2_. Fetal cardiovascular data transmitted wirelessly were recorded onto a laptop kept outside the hypoxic chamber laboratory. In this way, this system permitted continuous in vivo recording of fetal cardiovascular function without disturbing the animal’s environment and maintaining exposure to chronic hypoxia. Pregnancies allocated to the normoxia group were housed in a barn in floor pens with the same floor area as the hypoxic chambers. Both the normoxia and chronic hypoxia groups of ewes were also fed daily the same bespoke maintenance diet made up of concentrate pellets and hay (40 g nuts/kg and 3 g hay/kg; Manor Farm Feeds Ltd; Oakham, Leicestershire, United Kingdom) to facilitate the monitoring of maternal food intake.

### Daily Blood Sampling Regimen and Analysis

Samples (0.3 mL) of maternal and fetal arterial blood were taken daily to determine health and measuring arterial blood gas, acid-base and metabolic status, as before.^5,6,15–19^ Blood gas and acid base values were measured using an ABL5 blood gas analyser (Radiometer; Copenhagen, Denmark; maternal measurements corrected to 38°C, fetal measurements corrected to 39.5°C). Values for percentage saturation of hemoglobin with oxygen (Sat Hb) and for the concentration of hemoglobin in blood (Hb) were determined using a hemoximeter (OSM3; Radiometer). Blood glucose and lactate concentrations were measured using an automated analyser (Yellow Springs 2300 Stat Plus Glucose/Lactate Analyser; YSI, Ltd, Farnborough, United Kingdom). Values for hematocrit were obtained in duplicate using a microhematocrit centrifuge (Hawksley, United Kingdom).

### Pressor and Peripheral Vasopressor Responses to the α_1_-Adrenergic Agonist Phenylephrine

On the 10th day of exposure to normoxia or chronic hypoxia, fetuses were treated intravenously with bolus doses of phenylephrine (5, 12.5, 25, 37.5, and 50 μg; Sigma, United Kingdom) administered in random order (Figure S2). The dose regimen was adapted from previous experiments in our laboratory that resulted in dose-dependent changes in cardiovascular function in late gestation fetal sheep.^19^ For chronic hypoxic pregnancies, the fetal phenylephrine doses were administered while the ewes remained inside the hypoxic chambers. For normoxic pregnancies, the fetal phenylephrine doses were administered while the ewes were in a metabolic crate, inside a laboratory. Doses were given in random order when cardiovascular variables were at stable baseline. At least 10 minutes between doses were allowed for cardiovascular data to return to baseline. Maximal changes from baseline for fetal arterial blood pressure, fetal heart rate, femoral blood flow, and femoral vascular resistance were then calculated to each dose of phenylephrine, as before.^19,20^ Cardiovascular function returned to baseline conditions within 1 hour after the last dose of phenylephrine in all fetuses.

### Acute Hypotension Challenge

The day after dose responses, fetuses were exposed to an acute hypotension challenge (Figure S2). This consisted of 10 minutes of baseline, 10 minutes of hypotension, and 10 minutes of recovery. For chronic hypoxic pregnancies, the acute hypotension challenge occurred while inside the hypoxic chambers. For normoxic pregnancies, the acute hypotension challenge occurred while the animal was in a metabolic crate, inside a laboratory. Acute hypotension was induced via intravenous infusion of sodium nitroprusside (Sigma, United Kingdom, 50 μg/kg per minute). The dosing regimen of nitroprusside was adapted from past experiments in our laboratory, which achieved a significant reduction in arterial blood pressure that triggered compensatory cardiovascular responses.^20–22^

### Cardiac and Peripheral Vasomotor Baroreflex Responses to Acute Hypotension

Minute by minute average values for fetal arterial blood pressure, fetal heart rate, and femoral blood were downloaded continuously throughout the acute hypotension experimental protocol and imported into an Excel spreadsheet. Minute by minute average values for fetal femoral vascular resistance were then calculated using Ohm’s approximation and dividing arterial blood pressure (corrected for amniotic pressure) by femoral blood flow, as before.^16–19^ Baroreflex analysis was performed by plotting the heart rate against mean arterial blood pressure for each fetus in response to phenylephrine and nitroprusside. The slope of the linear relationship between changes in heart rate and in arterial blood pressure in response to phenylephrine was used to determine the vagal dominant contribution to the fetal cardiac baroreflex response.^22^

### Fetal Plasma Catecholamine Response to Acute Hypotension

Paired fetal carotid and femoral arterial blood samples (4 mL) were taken during baseline (−10, −5 minutes), during acute hypotension (+5, +10 min), and 10 minutes after recovery from hypotension (+20 min). These samples were used to determine blood gas and metabolic status, as before, and for analysis of fetal plasma noradrenaline and adrenaline concentrations (femoral sample only). For the catecholamine assay, blood was dispensed into EDTA-treated tubes, centrifuged for plasma extraction, and frozen at −80°C until analysis within 3 months of collection, as described before.^23,24^ An ELISA kit was used to determine the concentration of fetal plasma noradrenaline and adrenaline, in duplicate, following the manufacturer’s instructions (KA1877, Abnova, Taipei, Germany). For noradrenaline, the inter- and intraassay coefficients of variation were 12.8% and 14.6%, respectively, and the lower limit of detection was 0.05 ng/mL. For adrenaline, the inter- and intraassay coefficients of variation were 9.7% and 15.7%, respectively, and the lower limit of detection was 0.01 ng/mL.

### Oxygen and Glucose Delivery in the Carotid and Femoral Vascular Beds During Acute Hypotension

Fetal arterial blood oxygen content (C_a_O_2_) and regional changes in oxygen delivery (O_2,del_) were calculated according to Equations 1 and 2, as before^5,25^:


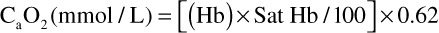
(1)


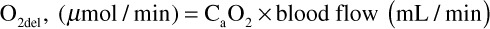
(2)

where (Hb) (g/dL) is the blood concentration of hemoglobin, Sat Hb (%) is the percentage oxygen saturation of hemoglobin and where 1 molecule of Hb (M.W. 64 450) binds 4 molecules of oxygen. The contribution of oxygen dissolved in plasma is regarded as negligible.^26^ Values for oxygen and for glucose delivery to the fetal carotid and femoral vascular beds were then calculated using Equations 3 and 4, respectively:


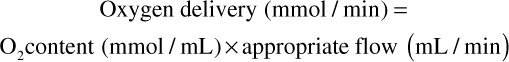
(3)


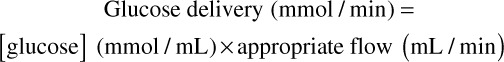
(4)

After the end of all experiments, all animals were transferred to the postmortem laboratory and humanely killed by overdose of sodium pentobarbitone (0.4 mL/kg IV Pentoject; Animal Ltd, York, United Kingdom). The fetus was weighed and the placement of flow probes and catheters was verified.

### Statistical Analysis

Appropriate power calculations derived from previous data sets were performed to determine the minimum sample size required to achieve statistical significance.^16–19,23–27^ The experiments were completed within one experimental season, and scientists measuring outcomes were blinded to treatments. All data are expressed as mean±SEM. Cardiovascular, endocrine, and metabolic data were analyzed using 2-way ANOVA, with repeated measures where appropriate or with the Student *t* test for unpaired data. When a significant interaction between main effects occurred, differences were compared using the Tukey or Student Newman Keuls post hoc tests. For all comparisons, statistical significance was accepted when *P*<0.05.

## Results

### Maternal and Fetal Health During Baseline and Exposure to Chronic Hypoxia

Basal maternal daily food consumption was not different between groups, and exposure to chronic hypoxia of this duration did not affect maternal food intake^5^ or fetal body weight when measured at post mortem (normoxia: 3.05±0.30 versus chronic hypoxia: 3.21±0.30 kg, *P*=NS).

**Figure 1.**
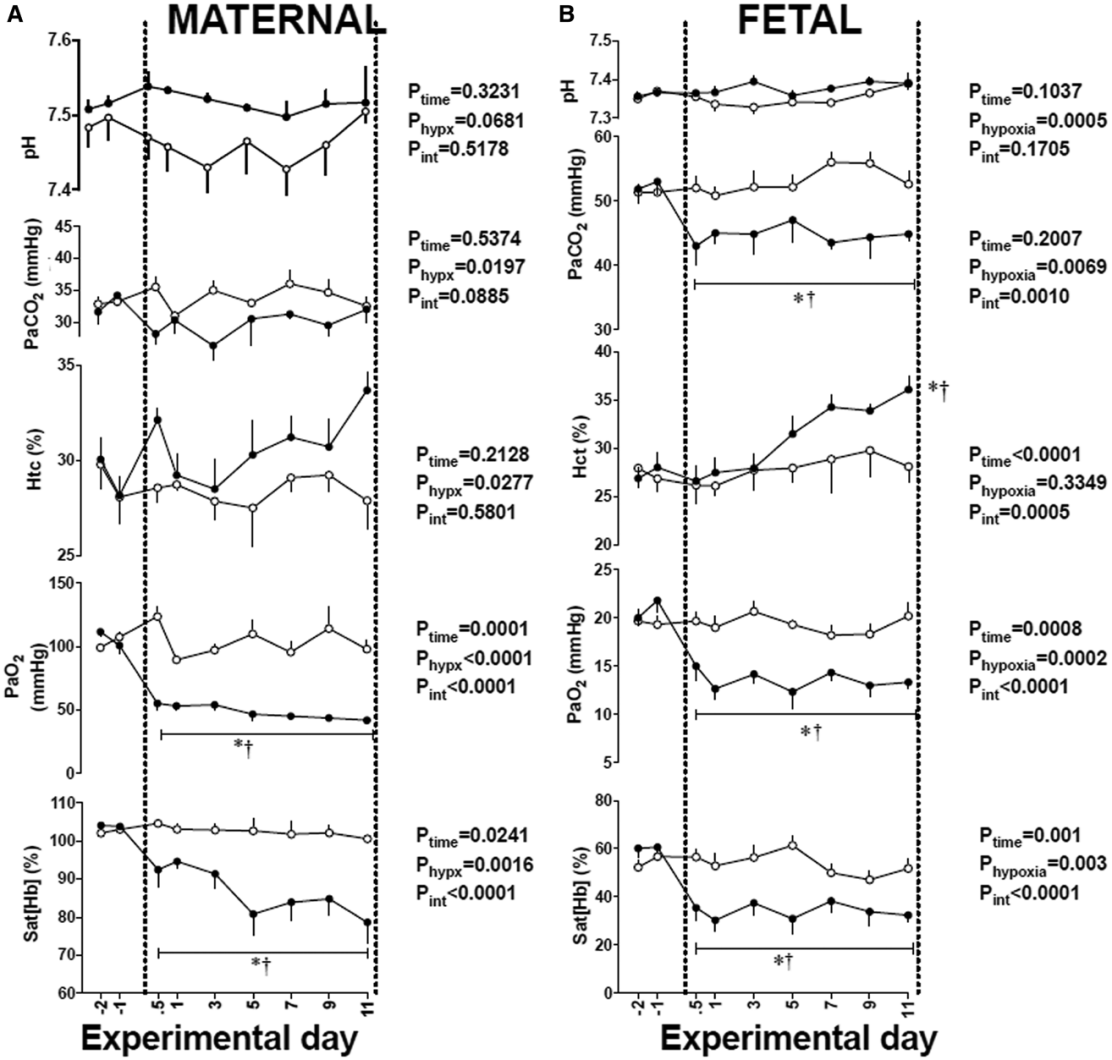
Maternal and fetal blood gases during chronic hypoxia. Values are mean±SEM. for the maternal (**A**) and fetal (**B**) arterial blood gas status in sheep undergoing normoxic (○, n=6) or chronic hypoxic (•, n=6) pregnancy. The results of the 2-way RM ANOVA for main effects and interactions are shown. When a significant (*P*<0.05) interaction between main effects occurred, differences were compared using the Tukey post hoc test. * indicates a significant effect of time compared with baseline; † indicates a significant effect of treatment compared with normoxic pregnancy. Htc indicates hematocrit; PaO_2_, arterial O_2_ partial pressure; PaCO_2_, arterial CO_2_ partial pressure; pH, arterial pH; and Sat [Hb], percentage saturation of hemoglobin.

Basal values for maternal arterial blood gas, acid-base, and metabolic status were not different between groups, and they were within the normal range for Welsh Mountain ewes^28^; Figure 1A. Ewes exposed to chronic hypoxia had a significant reduction in the partial pressure of arterial oxygen (mean±SEM: 106.4±3.7 to 47.3±1.9 mm Hg) and in arterial oxygen saturation (mean±SEM: 78.6±5.6%) compared with controls (100.6±0.5%) and their own baseline (*P*<0.05; Figure 1A). Further, ewes exposed to chronic hypoxia had significantly elevated hematocrit by the end of exposure (mean±SEM: 33.7±1.0 versus 27.9±01.5%). There was no significant change between groups in maternal arterial pH or partial pressure of arterial carbon dioxide (Figure 1A).

Basal values for fetal arterial blood gas, acid-base, and metabolic status were similar between groups and were within the normal range for Welsh Mountain singleton sheep fetuses at this stage of gestation^16–19,29^; Figure 1B). Fetuses exposed to chronic hypoxia had a significant reduction from baseline in the partial pressure of arterial oxygen (mean±SEM: 20.8±0.7 to 12.3±1.0 mm Hg) and arterial oxygen saturation (mean±SEM: 60.4±2.1 to 34.6±2.7%, *P*<0.05, Figure 1B). Fetuses exposed to chronic hypoxia also had significantly elevated hematocrit by the end of exposure (mean±SEM: 36.6±1.5 versus 28.2±2.0, *P*<0.05). There was no change between groups in fetal arterial pH; however, chronically hypoxic fetuses showed a significant fall in the partial pressure of arterial carbon dioxide (Figure 1B). Changes in values for fetal arterial blood pressure, fetal heart rate, fetal carotid blood flow, and fetal femoral blood flow during normoxia or chronic hypoxia have previously been reported.^5^ In brief, relative to controls, fetuses which were chronically hypoxic showed a smaller increase in arterial blood pressure and a delayed fall in fetal heart rate with advancing gestation. Further, measurement of blood flow in the carotid and femoral circulation revealed sustained increases during the period of chronic hypoxia.^5^

### Fetal Basal Blood Gas, Acid-Base Status, and Cardiovascular Function Before Acute Experiments

On day 10 of exposure, before the phenylephrine dose response experiment, chronically hypoxic fetuses remained with significantly lower values for PaO_2_, PaCO_2_, and SatHb, and significantly elevated values for hematocrit relative to normoxic fetuses (Table S1). Chronically hypoxic fetuses also had significantly reduced basal femoral blood flow and heart rate compared with normoxic fetuses (*P*<0.05; Table S1). Basal values for other fetal metabolic or cardiovascular variables before the phenylephrine dose response experiment were not different between groups (Table S1).

### Pressor and Femoral Vasopressor Responses to the α_1_-Adrenergic Agonist Phenylephrine

**Figure 2.**
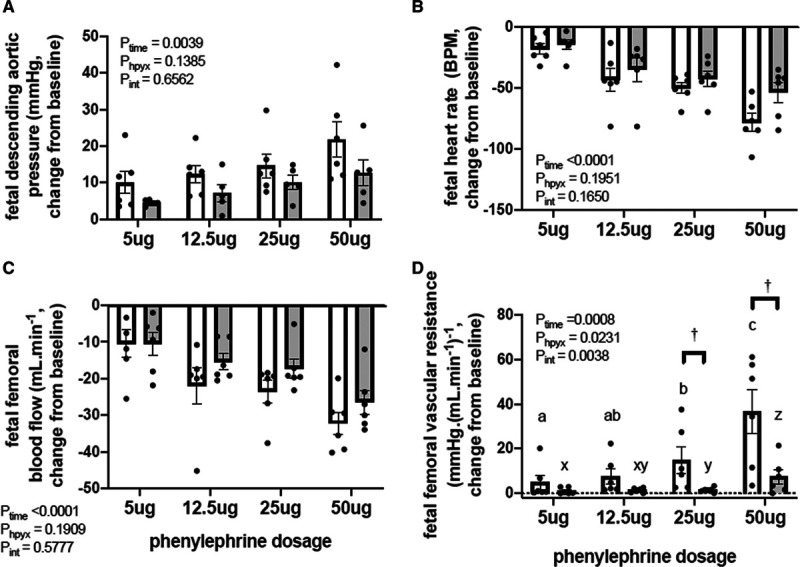
Pressor and femoral vasopressor responses to phenylephrine in the chronically hypoxic fetus. Values are the mean±SEM for the change from baseline in fetal arterial blood pressure (**A**), heart rate (**B**), femoral blood flow (**C**), and femoral vascular resistance (**D**) in response to increasing doses of phenylephrine (5, 12.5, 25, and 50 μg) in normoxic (open bars, n=6) and chronically hypoxic (gray bars, n=6) fetuses. The results of the 2-way RM ANOVA for main effects and interactions are shown. When a significant (*P*<0.05) interaction between main effects occurred, differences were compared using the Newman-Keuls post hoc test. Different letters indicate a significant effect of dose within groups (normoxic a–c, hypoxic x–z); † indicates a significant effect of treatment compared with normoxic pregnancy.

In normoxic fetuses, exogenous intravenous treatment with bolus doses of phenylephrine lead to dose-dependent increments from baseline in fetal arterial blood pressure and in fetal femoral vascular resistance, and in dose-dependent decrements from baseline in fetal heart rate and in fetal femoral blood flow (Figure 2A through 2D). In chronically hypoxic fetuses, the increments in fetal femoral vascular resistance were markedly diminished (Figure 2D). In particular, chronically hypoxic fetuses had significant blunting of the femoral vascular resistance response to higher doses of phenylephrine (25 μg H:1.5±0.4 versus N: 14.9±6.0 and 50 μg H:7.5±3.2 versus N:36.5±10.0 mm Hg/[mL/minute]; *P*<0.05, Figure 2D).

### Fetal Cardiovascular Response to Acute Hypotension and Fetal Cardiac Baroreflex Function

**Figure 3.**
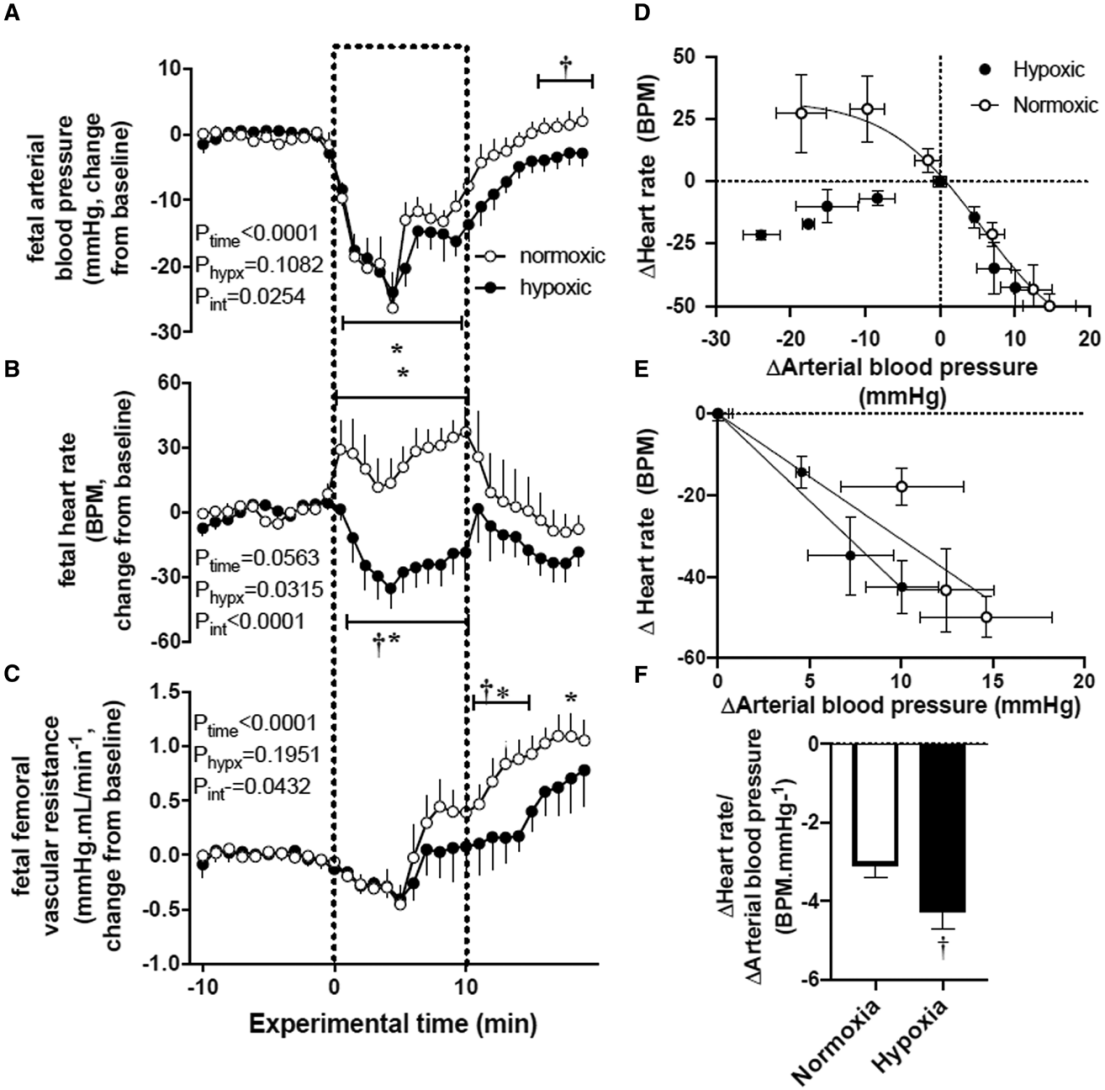
Cardiovascular responses to acute hypotension and cardiac baroreflex function in the chronically hypoxic fetus. **A–C**, Show the mean±SEM values for the change from baseline in arterial blood pressure (**A**), heart rate (**B**), and femoral vascular resistance (**C**) in normoxic (○, n=6) or chronically hypoxic (•, n=6) fetuses during the acute hypotension experiment. Acute hypotension (dashed box) was induced for 10 min by fetal intravenous infusion with sodium nitroprusside (2.5 mg/kg per min). **D–F**, Show an analysis of cardiac baroreflex function. The fetal arterial blood pressure-heart rate relationship was constructed by plotting the change from baseline in both variables mean±SEM (x and y) in response to sodium nitroprusside or to fetal treatment with increasing doses of phenylephrine (**D**). The vagal dominant component of the cardiac baroreflex is expanded in (**E**; mean±SEM. for x and y). **F**, Shows the mean±SEM values for an analysis of slopes for the vagal dominant component of the cardiac baroreflex. The results of the 2-way RM ANOVA for main effects and interactions for **A–C** are shown. When a significant (*P*<0.05) interaction between main effects occurred, differences were compared using the Tukey post hoc test. Groups in (**F**) were compared by the Student *t* test for unpaired data. *indicates a significant effect of time compared with baseline; †indicates a significant effect of treatment compared with normoxic pregnancy.

In normoxic fetuses, nitroprusside infusion resulted in a significant fall in arterial blood pressure from baseline (Table S1 and Figure 3A). The decrease in arterial blood pressure (Δ from baseline −26.3±5.2 mm Hg) was accompanied by a significant increase in fetal heart rate (Table S1 and Figure 3B, Δ from baseline 33.8±9.4 bpm) and in fetal femoral vascular resistance (Figure 3C, Δ from baseline 0.33±0.2 mm Hg/[mL/minute]; both *P*<0.05). The increase in fetal femoral vascular resistance occurred in parallel with a return in fetal arterial blood pressure toward baseline. During recovery, while femoral vascular resistance continued to increase, fetal arterial blood pressure and fetal heart rate returned to basal levels in normoxic fetuses (Figure 3). In marked contrast, in chronically hypoxic fetuses, although the fall in fetal arterial blood pressure was similar (Δ baseline −24.0±2.5 mm Hg), in these fetuses, fetal heart rate did not increase but decreased (Table S1; Figure 3B), and the increase in femoral vascular resistance during acute hypotension and recovery was significantly diminished compared with normoxic fetuses (Figure 3C). During recovery, chronically hypoxic fetus failed to return fetal arterial blood pressure to basal levels, maintaining a significantly reduced arterial blood pressure compared with normoxic fetuses by the end of the experiment (Δ from baseline −2.9±1.8 versus 2.0±2.0 mm Hg, *P*<0.05; Figure 3A). The arterial blood pressure-heart rate relationship in normoxic fetuses showed a normal cardiac baroreflex function with a traditional reverse sigmoidal plot for cardiac autonomic stimulation (Figure 3D). In marked contrast, the shape of the sympathetic quadrant of the cardiac baroreflex relationship depicting the changes in heart rate in response to decrements in arterial blood pressure induced by fetal treatment with nitroprusside was reversed in chronically hypoxic fetuses (Figure 3D). Calculation of the slope of the linear plot between changes in fetal arterial blood pressure and heart rate in response to phenylephrine in the vagal quadrant component of the cardiac baroreflex revealed a steeper relationship in chronically hypoxic fetuses relative to controls (Figure 3E and 3F).

### Fetal Plasma Catecholamine Response to Acute Hypotension

**Figure 4.**
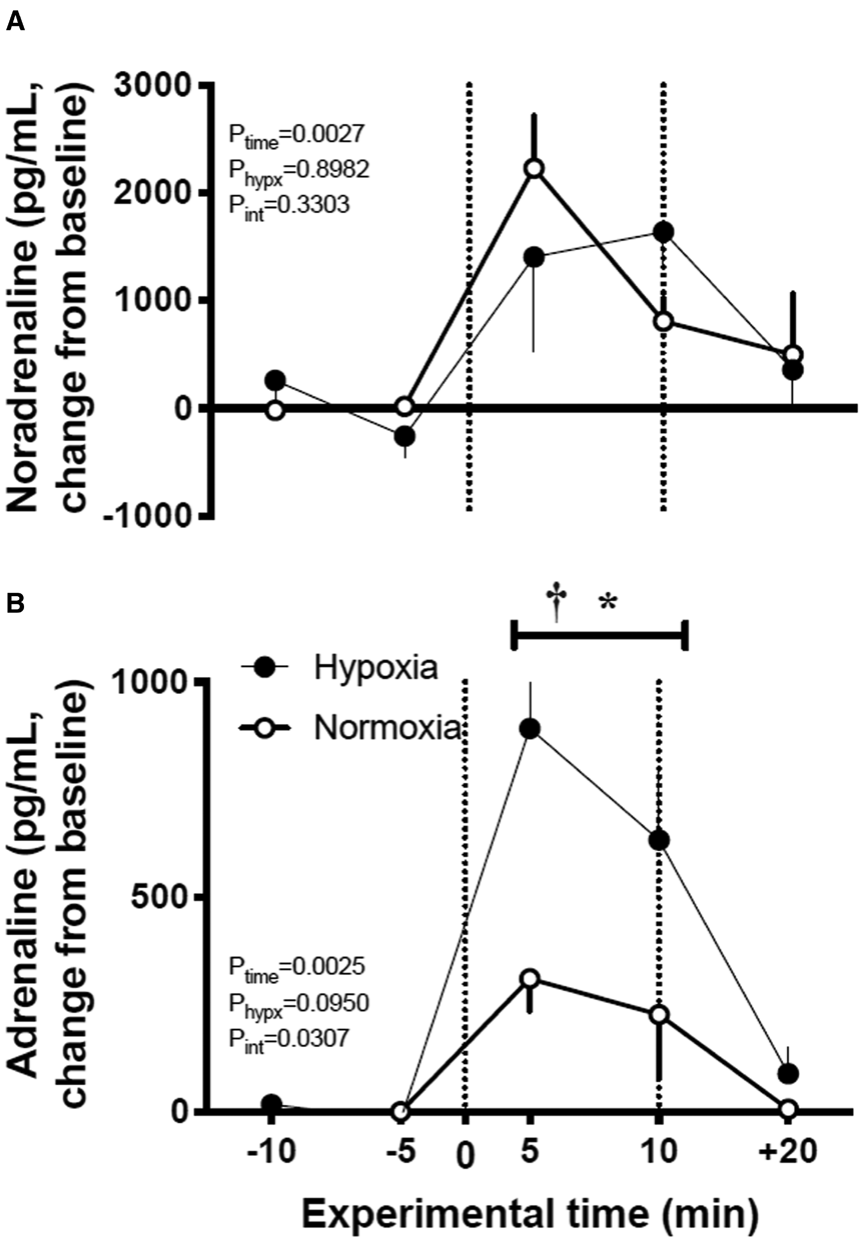
Plasma catecholamine response to acute hypotension in the chronically hypoxic fetus. Values are mean±SEM for the change from baseline in noradrenaline (**A**) and adrenaline (**B**) in normoxic (○, n=6) or chronically hypoxic (•, n=6) fetuses during the acute hypotension experiment. Acute hypotension (dashed box) was induced for 10 min by fetal intravenous infusion with sodium nitroprusside (2.5 mg/kg per min). Plasma samples were taken during baseline (−10 and −5 min), during hypotension (+5 and +10 min) and after 10 min of recovery (+20 min). The results of the 2-way RM ANOVA for main effects and interactions are shown. When a significant (*P*<0.05) interaction between main effects occurred, differences were compared using the Tukey post hoc test. *indicates a significant effect of time compared with baseline; †indicates a significant effect of treatment compared with normoxic pregnancy.

Exposure to chronic hypoxia did not affect fetal plasma catecholamine concentrations during baseline. Therefore, values for fetal basal plasma catecholamines concentrations before the acute hypotension experiment were not significantly different between normoxic (noradrenaline: 841.8±207; adrenaline: 66.4±4 pg/mL) and chronically hypoxic (noradrenaline: 1170±296; adrenaline: 88.6±14 pg/mL) fetuses. During acute hypotension, fetal plasma noradrenaline concentrations increased in a similar manner in both normoxic (Δ baseline 2229±486 pg/mL) and chronically hypoxic (Δ baseline 1403±874 pg/mL; Figure 4A) fetuses. In contrast, the increase in fetal plasma adrenaline concentrations during acute hypotension was significantly enhanced in chronically hypoxic fetuses compared with normoxic controls (Δ baseline H: 711±329 versus N: 310±77 pg/mL, *P*<0.05; Figure 4B). Both noradrenaline and adrenaline concentrations returned toward baseline levels in all fetuses during the recovery period.

### Changes in Fetal Oxygen and Glucose Delivery in the Carotid and Femoral Vascular Beds

**Figure 5.**
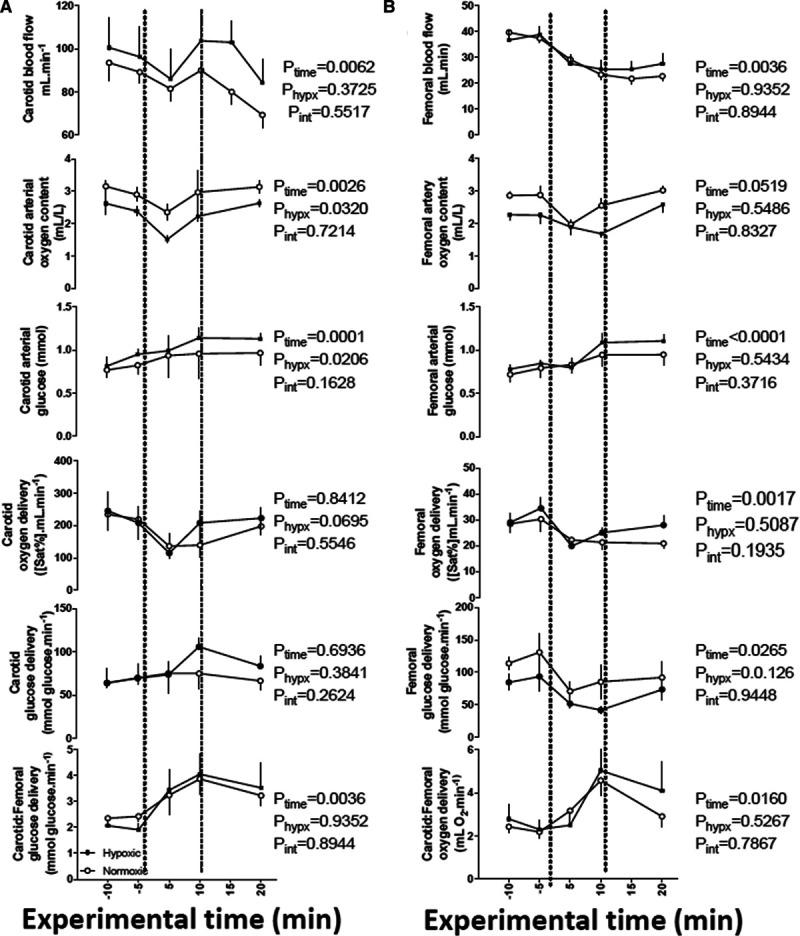
Changes in oxygen and glucose delivery in the carotid and femoral vascular beds during acute hypotension in the chronically hypoxic fetus. Values are mean±SEM for blood flow, oxygen content, blood glucose concentration, oxygen delivery, and glucose delivery in the carotid (**A**) and femoral (**B**) vascular beds. The ratio of oxygen and glucose delivery in the carotid relative to the femoral vascular beds is also calculated. Groups are normoxic (○, n=6) or chronically hypoxic (•, n=6) fetuses. Acute hypotension (dashed box) was induced for 10 min by fetal intravenous infusion with sodium nitroprusside (2.5 mg/kg per min). The results of the 2-way RM ANOVA for main effects and interactions are shown. There are no significant interactions between main effects.

During acute hypotension, fetal pH and arterial blood gases were maintained at basal levels in both normoxic and chronically hypoxic fetuses (Figure S3). During acute hypotension, carotid blood flow was better maintained than femoral blood flow in both groups of fetuses. However, there were no significant differences from baseline or between groups in either carotid or femoral blood flow (Figure 5A and 5B). During acute hypotension, oxygen content was similar in both carotid and femoral vascular beds in chronically hypoxic fetuses compared with normoxic fetuses (Figure 5A and 5B). In contrast, during acute hypotension, blood glucose concentrations increased in both normoxic and chronically hypoxic fetuses, although this only reached significance in both groups in the femoral and not carotid vascular bed (Figure 5A and 5B). During acute hypotension, oxygen and glucose delivery to both carotid and femoral vascular beds was maintained in normoxic and chronically hypoxic fetuses (Figure 5A and 5B). When the ratio of oxygen and glucose delivery in the carotid relative to the femoral vascular bed was calculated, both normoxic and chronically hypoxic fetuses showed a significant increase from baseline of similar magnitude in this ratio for both oxygen and glucose delivery during acute hypotension (Figure 5A and 5B). During recovery, blood glucose in the femoral vascular bed remained similarly elevated from baseline in normoxic and chronically hypoxic fetuses (Figure 5B), and the ratio of oxygen and glucose delivery in the carotid relative to the femoral vascular bed returned towards baseline in both groups (Figure 5A and 5B).

**Figure 6.**
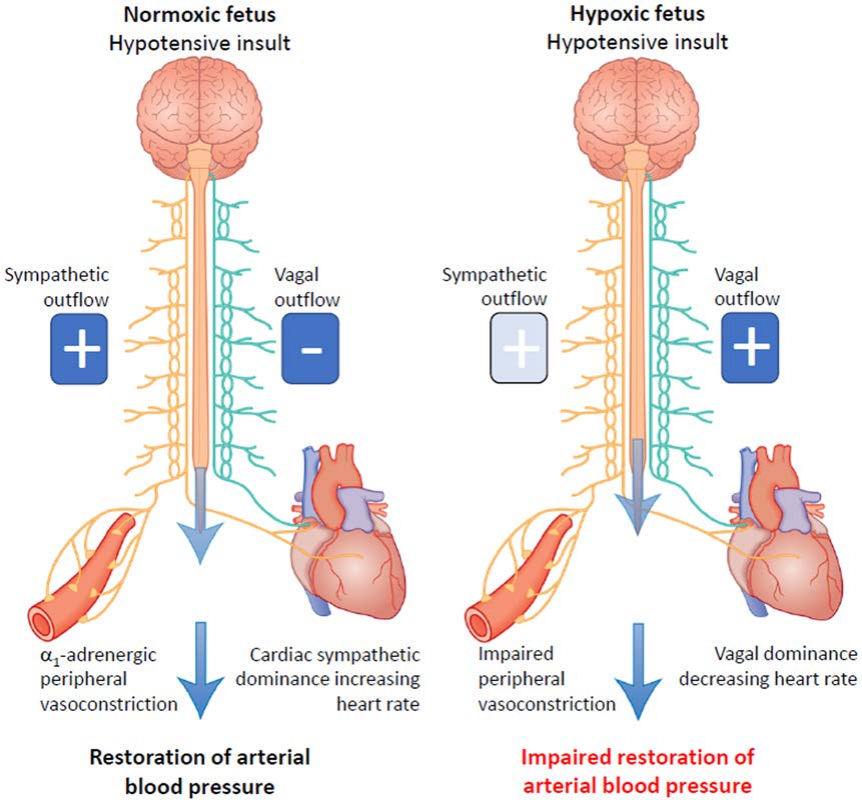
Summary figure. During acute hypotension, the normoxic fetus in healthy pregnancy activates cardiac and vasomotor baroreflex responses to restore blood pressure homeostasis. This involves baroreflex alteration in autonomic outflow via the brain stem. Increased sympathetic outflow coupled with vagal withdrawal to the heart promotes cardiac sympathetic dominant effects, which increase heart rate and contractility via β_1_-adrenergic receptor pathways. Enhanced sympathetic outflow to the arterioles increases peripheral vascular resistance via a1-adrenergic receptor pathways. Enhanced sympathetic outflow to the veins increases venous return via a1-adrenergic receptor pathways. The increase in venous return, coupled with increased myocardial contractility lead to an increase in cardiac output. The increase in peripheral vascular resistance together with the increase in cardiac output restores fetal arterial blood pressure back to baseline. In the chronically hypoxic fetus, baroreflex-induced increases in heart rate do not occur. Instead, there is a fall in heart rate because of increased vagal dominant effects. In addition, responsiveness to α_1_-adrenergic receptor pathway stimulation is blunted in the peripheral vasculature. Combined, these effects of chronic hypoxia on the heart and circulation weaken the fetal cardiovascular defense to acute hypotension in the chronically hypoxic fetus, providing a mechanistic link underlying its greater susceptibility to a second hit.

## Discussion

These data show that chronic hypoxia has a significant impact on the fetal cardiovascular defense to a superimposed acute hypotensive challenge, supporting the hypothesis tested in this study. Markedly impaired baroreflex responses, vagal dominant regulation of the fetal heart during acute hypotension, and diminished constrictor reactivity in the peripheral vasculature to α_1_-adrenergic stimulation provide in vivo evidence of underlying mechanisms explaining the altered cardiovascular responses in the chronically hypoxic fetus. However, the plasma catecholamine response to acute hypotension was not diminished; in fact, the fetal plasma adrenaline response to acute hypotensive stress was markedly enhanced in the chronically hypoxic relative to the normoxic fetus.

Chronic hypoxia induces the activation of the HIF (hypoxia-inducible factor), which regulates the expression of hundreds of genes including erythropoietin or *EPO*, which enhances red blood cell production, and this effect is measured by an increase in hematocrit.^30^ We show that exposure pregnant ewes to ca 10% O_2_ for 10 days led to an increase in maternal and fetal hematocrit levels, confirming HIF activation. This level of chronic hypoxia was chosen as it reduces the fetal PaO_2_ in the descending aorta from normal values of ca 20 mm Hg to values of 12 to 14 mm Hg. This magnitude of fetal chronic hypoxia is important because such fetal PaO_2_ values are similar to those measured by cordocentesis in human fetuses affected by growth restriction, as reported in pioneering clinical studies.^31–33^ In the present study, there was also a reduction in fetal PaCO_2_ in response to chronic hypoxia. This may indicate alterations in the rate of fetal metabolism in the presence of hypoxia, minimizing fetal oxygen consumption with a consequent fall in fetal CO_2_ production.^5^ Importantly, this level of maternal hypoxia did not affect maternal food intake. Therefore, the adverse effects on the fetal cardiovascular capacity to respond to acute hypotension measured in the present study are those of isolated chronic hypoxia, of levels that are human clinically relevant.

To address mechanisms explaining the weaker peripheral vasoconstrictor response and the reversed heart rate response to acute hypotension in the chronically hypoxic fetus, we determined fetal in vivo pressor and femoral vasopressor responses to exogenous bolus doses of the α_1_-adrenergic receptor agonist phenylephrine, we constructed cardiac baroreflex function curves, and we measured the fetal in vivo endocrine catecholamine response during the acute hypotension challenge. The dose-response experiment in the present study revealed that femoral vasopressor responses to α_1_-adrenergic receptor stimulation are markedly blunted in the chronically hypoxic fetus. The cardiac baroreflex analysis showed an absent sympathetic component altogether with enhanced vagal sensitivity regulating cardiac function. Measurement of plasma catecholamines during the acute hypotension experiment further revealed that the fetal plasma catecholamine response is not suppressed; rather, the fetal plasma adrenaline response to acute hypotension is enhanced in the chronically hypoxic fetus compared with the normoxic fetus. Combined, therefore, these data show that reduced plasma catecholamine responses could not explain the cardiovascular deficits to acute hypotension. Instead, chronic hypoxia impaired fetal femoral vasopressor responses mediated via α_1_-adrenergic receptor stimulation and switched the balance of the autonomic regulation of cardiac function towards vagal dominance during acute hypotension. Clinically, fetal hypotension can be linked with profound fetal hypoxia.^34^ Therefore, one could argue that the fall in heart rate during acute hypotension in the chronically hypoxic fetus may result from carotid chemoreceptor reflex activation^3,17^ rather than a failed cardiac baroreflex. However, this idea is not supported, as the arterial blood gas data during acute hypotension in the present study show that PaO_2_ was not reduced from baseline during the acute hypotension challenge in the chronically hypoxic fetus.

In the human fetus, autonomic function can be assessed noninvasively by measurement of fetal heart rate variability (FHRV). Several investigators have reported that reduced FHRV may be associated with fetal growth restriction and fetal compromise.^14,15,35,36^ Therefore, computerized cardiotocography examination is recommended for antenatal surveillance of human fetuses with suspected placental insufficiency and chronic fetal hypoxia.^37^ A recent study in our laboratory using this model of chronic fetal hypoxia in sheep reported that FHRV is reduced by chronic hypoxia, predominantly because of dysregulation of the sympathetic control of the fetal heart.^15^ Another comprehensive study in sheep reported that chronic hypoxia increased binding of angiotensin receptors in the fetal brain stem, and this was associated with a fall in spontaneous baroreflex sensitivity and evidence of vagal dominant regulation of heart rate.^38^ Finally, an elegant series of studies using long-term exposure of ovine pregnancies to the chronic hypobaric hypoxia of life at high altitude have reported impaired signaling in both β_1_- and α_1_-adrenergic receptor pathways in the heart and vasculature, respectively, in chronically hypoxic fetal sheep.^39,40^ Combined, data from all these studies are consistent with the findings reported in the present investigation, indicating both alterations in the central autonomic regulation of cardiovascular function as well as changes in the reactivity of cardiovascular target organs to noradrenergic stimulation in the chronically hypoxic fetus. As several clinical studies have reported that the growth-restricted human infant displays compromised autonomic cardiovascular control as well as weak defenses to acute challenges,^8,10^ data in the present study support that these adverse effects in the growth restricted fetus are likely to be due to those induced by chronic fetal hypoxia alone. Reduction in indices of FHRV as a result of sympathetic suppression may therefore predict chronically hypoxic fetuses at increased risk of birth asphyxia during acute events and at increased risk of neonatal morbidity and mortality in humans. A reduction in overall FHRV may therefore provide a clinical biomarker indicating that autonomic dysregulation of fetal heart rate control has taken place in a fetus with suspected uteroplacental compromise.

Human clinical studies have also associated reductions in FHRV in growth restricted pregnancies with increased risk of blood pressure instability, predisposing the human infant to systemic hypoperfusion, thereby reducing oxygen and glucose delivery to the fetal brain.^8,41^ For this reason, it was of interest to measure changes in oxygen and glucose delivery to the fetal carotid and femoral vascular beds during acute hypotension in the present study. During acute stress, the fetal circulatory response redistributes the delivery of nutrients away from peripheral and toward essential vascular beds, such as those perfusing the brain.^2,3^ This brain sparing defense can be illustrated by calculating the ratio of oxygen and glucose delivery in the carotid relative to the femoral vascular beds during the episode of acute stress.^3,5^ Data in the present study show that maintenance of oxygen and glucose delivery toward the fetal brain was not altered in the chronically hypoxic relative to normoxic fetus.

In fetal life, the origin of the increase in both plasma adrenaline and noradrenaline during acute stressful conditions is the adrenal gland, rather than from sympathetic neuronal spill-over.^42^ The enhanced plasma adrenaline response to acute hypotensive stress in the chronically hypoxic fetus in the present study is consistent with reports of an enhanced catecholamines response to acute hypoxic stress in chronically hypoxic fetal sheep.^43^ Possible mechanisms driving an enhanced plasma adrenaline response to acute stress in the chronically hypoxic fetus may include a sensitized activation of the splanchnic nerve, as a major component of the increase in total catecholamine output from the fetal adrenal gland during acute stress is mediated via stimulation of the splanchnic nerves.^42,44^ In addition, chronic hypoxia may affect the expression and activity of catecholamine synthetic enzymes TH (tyrosine hydroxylase) and PNMT (phenylethanolamine N-methyltransferase; see study by Adams and McMillen^45^). The functional significance of the increase in plasma adrenaline in response to acute stress in the late gestation fetus is to inhibit insulin while stimulating glucagon, decrease glucose uptake by the fetal tissues, and mobilize the increased blood glucose levels, prioritizing glucose delivery to essential organs like the fetal brain.^46,47^

It is of interest that despite a weaker cardiac and vasomotor baroreflex response to acute hypotension and impaired peripheral vascular reactivity to α_1_-adrenergic agonists, oxygen and glucose delivery to the fetal brain was maintained during acute hypotensive stress in the chronically hypoxic fetus. It is possible that the enhanced fetal plasma adrenaline response in the chronically hypoxic fetus is an adaptive response, attempting to compensate for the poor cardiac sympathetic response while ensuring adequate delivery of glucose to the fetal brain during acute hypotension. Therefore, the chronically hypoxic fetus may adopt alternative defense mechanisms to maintain brain sparing, at least in response to short-term challenges in blood pressure homeostasis. The magnitude of the fall in fetal arterial blood pressure during acute hypotension in the present study is similar or even less severe than what might be expected to be induced by single or repeated umbilical cord compression in the clinical setting (see article by Bennet^34^). How the chronically hypoxic fetus would respond to episodes of similar or alternative forms of stress of greater severity and/or longer duration and/or increased frequency is clearly a rich avenue of future research.

There are several advantages and limitations of using sheep as the species of choice for such investigations in fetal cardiovascular physiology. An advantage is that sheep and humans share more similar prenatal developmental milestones of cardiovascular anatomy and physiology, in contrast to rodents which are born highly immature.^48,49^ In addition, the chronically instrumented fetal sheep preparation is the only established animal model that permits surgical implantation of catheters and flow probes for prolonged cardiovascular recording, yielding a physiologically robust, long-term preparation. This level of insight does not exist for any other species. Further, sheep and humans give birth primarily to singleton or twin offspring, in contrast to rodents and pigs that give birth to litters. Hence, the maternal metabolic investment in the pregnancy is likely more similar between sheep and humans, relative to many other species, as multiple pregnancies increase the maternal basal metabolic rate.^50^ In contrast, there are important limitations of the current study. One obvious one is cost of using sheep for long term studies relative to using rodents. The study design therefore controlled for, but it did not address sex differences. Limitations in cost also reduces the number of animals per group, thereby increasing the number of experimental comparisons that are likely underpowered for statistical analysis.

In conclusion, this study has permitted in vivo continuous recording of cardiovascular function in fetuses undergoing highly controlled chronic hypoxia and exposed to an acute secondary stressor, highlighting the strength of the conceptual advance to the field of study. We show that the chronically hypoxic fetus displays markedly altered cardiovascular responses to an acute hypotensive stress. Such effects may explain the well-known greater susceptibility of the human growth restricted fetus, presumed to be chronically hypoxic, to a second hit.

### Perspectives

This study shows what happens when a chronically hypoxic fetus is exposed to a further stressor in utero. Clinically, such a situation could arise in human pregnancy when a growth-restricted fetus is exposed to excessive uterine contractions in labor, acute cord compression, or an acute placental hemorrhage event. In the clinic, this can lead to acute decompensation of fetal cardiovascular defenses, failure to maintain fetal arterial blood pressure, culminating in severe hypoxic ischemic encephalopathy with subsequent fetal brain damage. This situation is difficult to recreate in vivo in an experimental animal model. Here, we have exploited novel technology recently available to induce a controlled period of chronic hypoxia in sheep pregnancy at levels of human clinical relevance and investigated the effects of a superimposed second hit in the form of an acute hypotensive challenge. We show that chronic hypoxia has a significant effect on the fetal cardiovascular defense responses to a second hit. This provides in vivo evidence of mechanisms linking the greater susceptibility of the chronically hypoxic fetus to superimposed stress and adds to the body of evidence behind the cause of brain damage after fetal growth restriction. Antenatal and intrapartum periods of hypoxia leading to collapse of fetal cardiovascular defenses are important causes of fetal brain damage and common grounds for litigation in many national health systems. Therefore, the data reported in this manuscript may help to diagnose the compromised fetus where uteroplacental dysfunction is suspected to try and improve neonatal outcome.

## Acknowledgments

We thank the staff of the University Biological Services at The Barcroft Centre, University of Cambridge. D.A. Giussani is the Professor of Developmental Cardiovascular Physiology & Medicine at the Department of Physiology Development & Neuroscience at the University of Cambridge, Professorial Fellow and Director of Studies in Medicine at Gonville & Caius College, a Lister Institute Fellow and a Royal Society Wolfson Research Merit Award Holder.

## Sources of Funding

This work was supported by the British Heart Foundation (RG/17/8/32924).

## Disclosures

License agreement 100395 CamDAS: Technology for simultaneous wireless recording of arterial blood pressure and blood flow in large animals. D.A. Giussani, Maastricht Instruments, The British Heart Foundation and Cambridge Enterprise. The other authors report no conflicts.

## Supplementary Material



## Novelty and Significance

### What Is New?

- The growth-restricted fetus, presumed to be chronically hypoxic, is at a 7-fold risk of acute complications later in gestation. However, the reason behind this is unknown.
- We show that chronic hypoxia substantially alters the fetal cardiovascular defense to a second hit, providing an explanation for this well-established vulnerability.

### What Is Relevant?

- Adverse conditions during pregnancy can trigger a fetal origin of increased disease risk in the offspring. This study shows that chronic hypoxia, one of the most common complications in human pregnancy, substantially affects fetal defense mechanisms, increasing a future risk of cardiovascular and neurodevelopmental dysfunction after birth.

### Summary

- Chronic hypoxia has a significant impact on the fetal cardiovascular defense to a second hit.
