# Supplementary material for: Altered Cardiovascular Defense to Hypotensive Stress in the Chronically Hypoxic Fetus
Source: Hypertension. 2020 Aug 31;76(4):1195–207. doi: 10.1161/HYPERTENSIONAHA.120.15384 (PMC7480941; doi:10.1161/HYPERTENSIONAHA.120.15384)
Supplement: Supplementary file 1 [file hyp-76-1195-s001.pdf]

DATA SUPPLEMENT

Altered Cardiovascular Defense to Hypotensive Stress in the Chronically Hypoxic Fetus

Beth J Allison[1], Kirsty L Brain[1], Youguo Niu[1], Andrew D Kane[1], Emilio A Herrera[1], Avnesh S Thakor[1,2], Kimberley J Botting[1], Christine M Cross[1], Nozomi Itani[1], Caroline J Shaw[1,3], Katie L Skeffington[1], Christian Beck[1] & Dino A Giussani[1,,4,5]

[1] Department of Physiology, Development & Neuroscience, University of Cambridge, Downing Street, Cambridge, CB2 3EG, UK

[2] Department of Radiology, Stanford University Medical Center, Palo Alto, CA, 94305, USA

[3] Institute of Reproductive and Developmental Biology, Imperial College, London W12 0HS, UK

[4] Cambridge Cardiovascular Strategic Research Initiative

[5] Cambridge Strategic Research Initiative in Reproduction

	pH	PaO_2 (mmHg)	$PaCO_2$ (mmHg)	Sat[Hb] (%)	Hct (%)	Glucose (mmol)	Lactate (mmol)	BP (mmHg)	HR (BMP)	FBF ($mL.min^{-1}$)	CBF ($mL.min^{-1}$)	Oxygen Content ($mmol.L^{-1}$)
N	7.38 ± 0.01	19.5 ± 1.06	53.0 ± 1.0	58.4 ± 6.1	28.1 ± 2.7	0.701 ± 0.10	1.16 ± 0.17	39.0 ± 3.5	164.6 ± 4.7	45.6 ± 2.3	92.9 ± 8.8	2.87 ± 0.13
H	7.41 ± 0.02	14.5 ± 0.4 †	41.5 ± 1.9†	39.3 ± 3.5†	35.1 ± 1.1†	0.810 ± 0.07	1.87 ± 0.38	37.5 ± 3.3	150.0 ± 3.9 †	36.7 ± 1.6†	97.4 ± 8.2	2.26 ± 0.14 †

Table S1. Basal fetal arterial blood gas, metabolic status and cardiovascular function prior to acute experiments. Values are mean±S.E.M. for fetal arterial blood pH, arterial partial pressure of oxygen (PaO_2), arterial partial pressure of carbon dioxide ($PaCO_2$), percentage saturation of haemoglobin with oxygen (Sat[Hb]), blood glucose and lactate concentrations, arterial blood pressure (BP), heart rate (HR), femoral blood flow (FBF) and carotid blood flow (CBF) in normoxic (N, n=6) or chronic hypoxic (H, n=6) fetuses prior to acute experiments. Significant differences (P<0.05) are: †, differences indicating a significant effect of treatment compared with normoxic pregnancy (Student's t test for unpaired data).

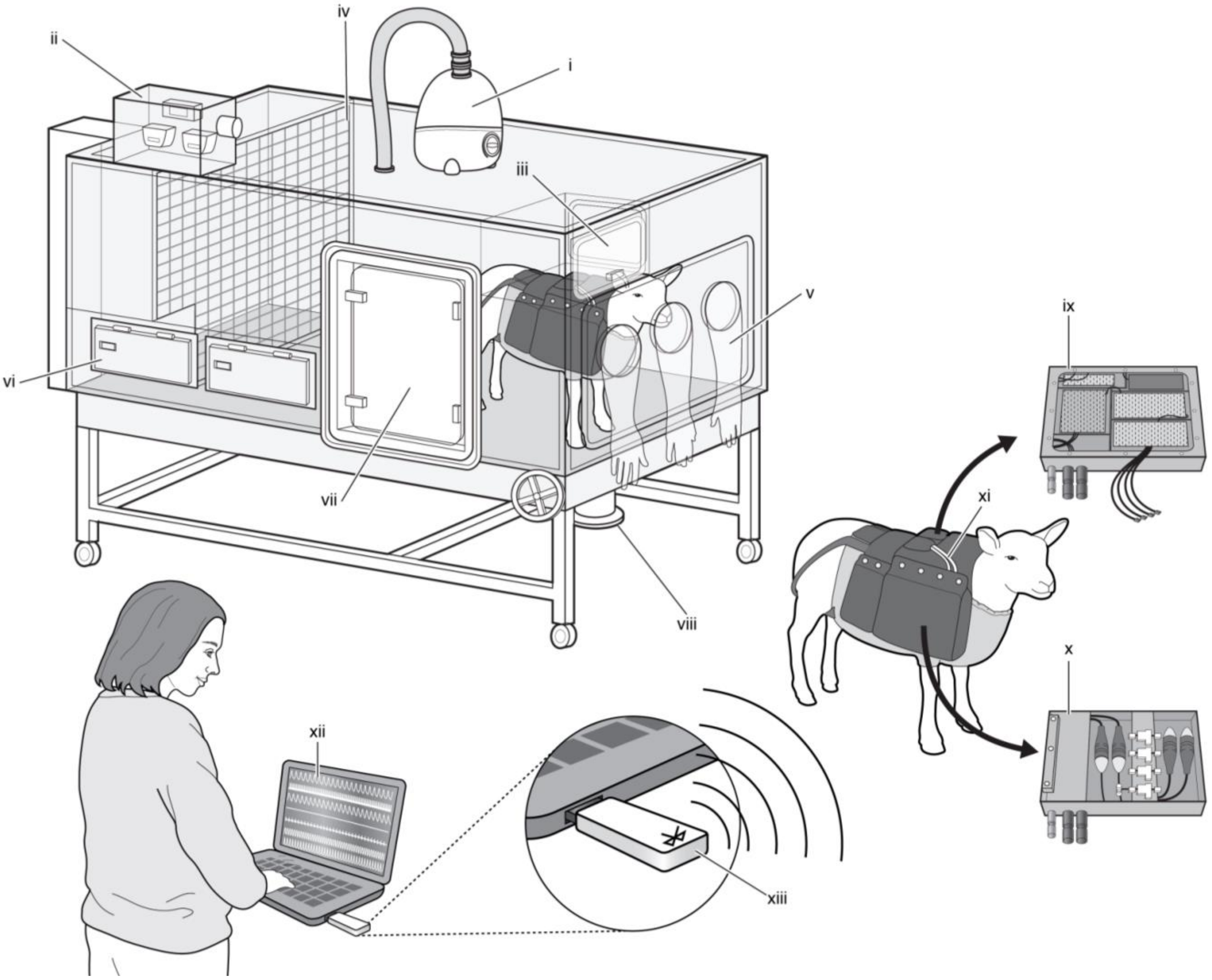

Figure S1. Hypoxic chambers and the camDAS system. Each chamber contained an electronic servo-controlled humidity cool steam injection system to maintain appropriate humidity to the inspirate (i). Ambient PO_2, PCO_2, humidity and temperature within each chamber were monitored via sensors (ii). For experimental procedures, each chamber had a double transfer port (iii) to internalise material and a manually operated sliding panel (iv) to bring the ewe into a position where daily sampling of blood could be achieved through glove compartments (v). Each chamber had a drinking bowl on continuous water supply and a rotating food compartment (vi) for determining food intake. A sealed transfer isolation cart could be attached to a side exit (vii) to couple chambers together for cleaning or for animal movement. Animal waste was collected through an outlet containing a sealing

valve (viii). The camDAS system was contained in a custom-made sheep jacket able to hold a miniaturized Transonic flow box (ix) on one side, and a pressure box (x) on the other. Cables (xi) connected the flow and pressure boxes to two battery packs able to power the system for >24 hours. Measurements made using the data acquisition were transmitted wirelessly to a laptop kept outside the chamber (xii) via Bluetooth technology (xiii), thereby allowing continuous monitoring of fetal cardiovascular function *in vivo* in the chronically hypoxic fetus. Reproduced with permission [5].

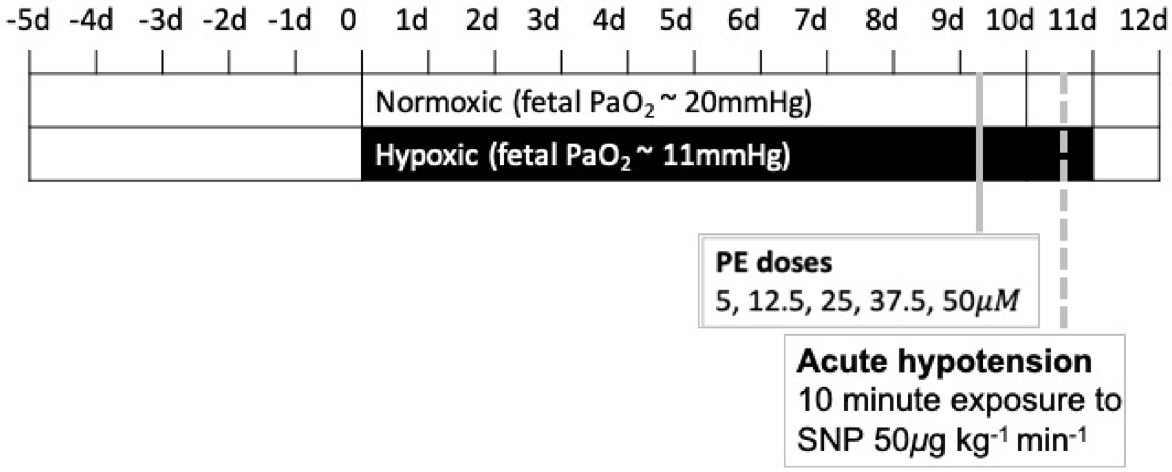

Figure S2. Experimental design. The experimental protocol consisted of 5 days (d) of baseline recording followed by either chronic normoxia (white bar, n=6) or chronic hypoxia (black bar, n=6, $\%PO_2$ ~10.5 mmHg). On day 10 of exposure, fetuses received increasing bolus doses of phenylephrine (PE; 5, 12.5, 25, 37.5 and 50µg) administered in random order. The following day, fetuses were exposed to an acute hypotension experiment via infusion of sodium nitroprusside (SNP; 2.5µg/kg/min).

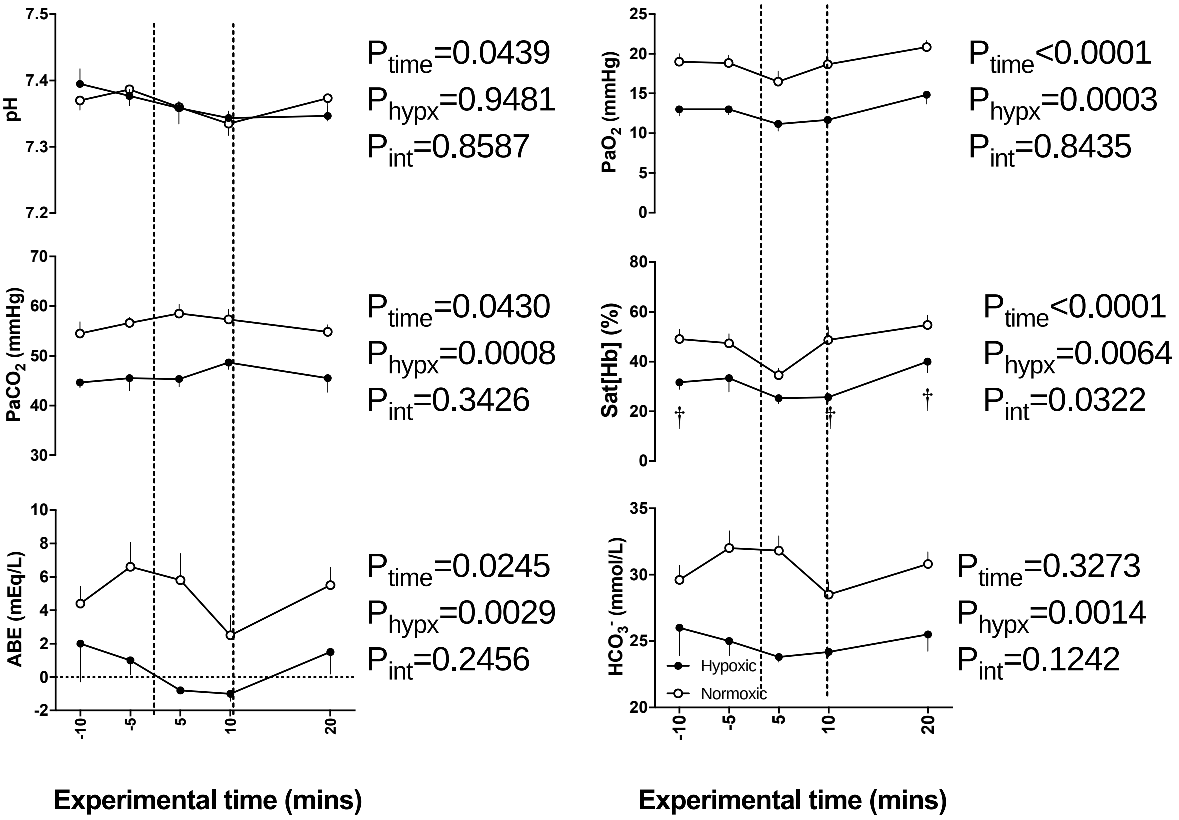

Figure S3. Arterial blood gas status during acute hypotension in the chronically hypoxic fetus. Values are mean±S.E.M. for arterial pH (A); $PaCO_2$, arterial CO_2 partial pressure (B); PaO_2, arterial O_2 partial pressure (C); Sat[Hb], percentage saturation of hemoglobin (D), ABE, Acid-base excess (E) and HCO_3^-, bicarbonate (F) in fetal sheep during acute hypotension in normoxic (O, n=6) or chronic hypoxic (●, n=6) pregnancy. The results of the two-way RM ANOVA for main effects and interactions are shown. When a significant (P<0.05) interaction between main effects occurred, differences were compared using the Tukey *post hoc* test. * indicates a significant effect of time compared with baseline; † indicates a significant effect of treatment compared with normoxic pregnancy.
