# Supplementary figures and images for: Altered Cardiovascular Defense to Hypotensive Stress in the Chronically Hypoxic Fetus
Source: Hypertension. 2020 Aug 31;76(4):1195–207. doi: 10.1161/HYPERTENSIONAHA.120.15384 (PMC7480941; doi:10.1161/HYPERTENSIONAHA.120.15384)

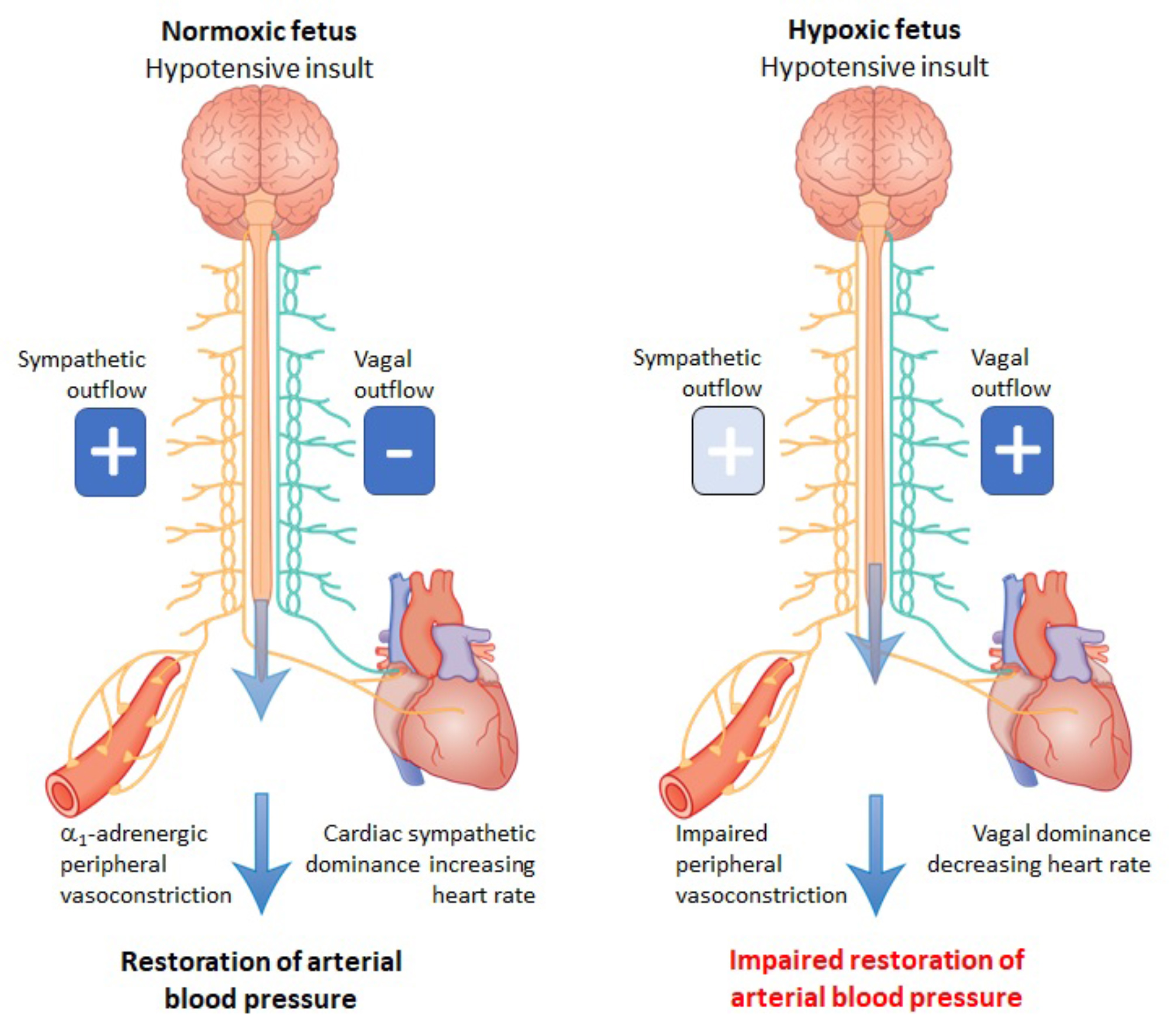

Supplement: Supplementary file 2 [file hyp-76-1195-s002.jpg]
