# Supplementary material for: Altered Cardiovascular Defense to Hypotensive Stress in the Chronically Hypoxic Fetus
Source: Hypertension. 2020 Aug 31;76(4):1195–207. doi: 10.1161/HYPERTENSIONAHA.120.15384 (PMC7480941; doi:10.1161/HYPERTENSIONAHA.120.15384)
Supplement: Supplementary file 3 [file hyp-76-1195-s003.pdf]

*** Short In Vivo Checklist**

AHA - Preclinical Animal Testing: Prevention of bias is important for experimental cardiovascular research. ***This short checklist must be completed, and the answers should be clearly presented in the manuscript as well.*** *The checklist will be used by reviewers and editors but will not be published. If a revision is requested, you will be required to complete at revision submission a more detailed checklist that will be published with the accepted article.*

This study involves animals:
Yes

Animals

Species, age, sex, strains, and sources of animals are described: Yes

Randomization

Randomization and allocation concealment were performed: Yes

Blinding

Blinding was performed: Yes

Inclusions and Exclusions (a)

Specific criteria for inclusions and exclusions are specified: Yes

Inclusions and Exclusions (b)

Criteria for inclusions and exclusions were set before the study: Yes

Reporting of Excluded Animals

All animals excluded after the randomization are reported: Yes

Statistical Methods

Statistical Methods are described: Yes

Date completed: 07/07/2020 12:19:31
User pid: 7421
